# pH-responsive interface conversion efficient oral drug delivery platform for alleviating inflammatory bowel disease

**DOI:** 10.3389/fchem.2024.1365880

**Published:** 2024-03-12

**Authors:** Yingying Zhao, Changqing Xu, Qing Liu, Xiaofei Lei, Li Deng, Fengyan Wang, Jing Yang

**Affiliations:** ^1^ Department of Gastroenterology, The First Affiliated Hospital of Shandong First Medical University & Shandong Provincial Qianfoshan Hospital, Shandong, China; ^2^ Department of Clinical Laboratory, Shanghai Gongli Hospital, The Second Military Medical University, Shanghai, China

**Keywords:** rough mesoporous silica, Mesalazine, oral delivery, colitis, pH-responsive

## Abstract

A key challenge for the effective treatment of intestinal diseases, including inflammatory bowel disease (IBD), is to develop an oral drug delivery system that can resist gastric acid erosion and efficiently release drugs after rapid entry into the intestine. In the present work, we developed oral composite nanoparticles (MSZ@PRHS) consisting of a rough mesoporous silica (RHS) loaded with Mesalazine (MSZ) and a CAP polymer membrane for targeted relief of inflammation in colitis. At the pH values of the simulated stomach and small intestine, the release rate of MSZ from MSZ@PRHS was low, while at the pH values of the simulated colon, the release rate of MSZ was high. In dextran sulfate sodium salt (DSS)-induced acute colitis mouse model, compared with oral administration of the drug Mesalazine in the equivalent solution form, oral administration of PRHS loaded with drug-loaded nanoparticles can significantly alleviate the symptoms of inflammatory bowel disease, and improve the therapeutic effect. We propose that the intestinal microenvironment provides an interface for nanocomposites switch and a promising drug delivery platform for the management and treatment of many intestinal diseases, where controlled drug release and prolonged residence time are required.

## 1 Introduction

Inflammatory bowel disease (IBD), including ulcerative colitis (UC) and Crohn’s disease (CD), is a chronic inflammatory disease characterized by accumulation of submucosal inflammatory cells and epithelial damage (Wright et al., 2018; Jin et al., 2021; Liu et al., 2022). The prevalence of IBD is increasing worldwide, which further increases the risk of its transformation into colon cancer (Ng et al., 2017). Currently, there is no complete cure for IBD, amino salicylates, steroids, immunosuppressants, and biologics are the most commonly used drugs for the treatment of IBD (Goren et al., 2020; Lee et al., 2020; Baumgart and Le Berre, 2021). Because many patients who are diagnosed with IBD require lifelong treatment to control symptoms, patients prefer oral administration over enema, subcutaneous injection, and intravenous injection in terms of compliance.

At present, the side effects caused by systemic drug exposure caused by frequent oral administration of traditional drug delivery systems are more serious. Therefore, the systemic absorption of these drugs must be reduced to reduce the incidence of adverse side effects. Traditional formulations for oral colon-specific drug delivery utilize specific physiological characteristics of the colonic region (e.g., pH, pressure, and zymogram) to trigger drug release. However, the physiological conditions vary from patient to patient and from stage to stage of IBD. Therefore, it is difficult to achieve the ideal therapeutic effect using conventional methods. With the continuous breakthrough in the understanding of the molecular pathophysiology of inflammatory bowel disease and the development of intelligent drug delivery materials, oral colon-specific drug delivery has broad prospects for the treatment of inflammatory bowel disease.

This study aims to assemble a pH-responsive nanoparticle (MSZ@PRHS), a safe and effective colon-targeted delivery system, and investigate its therapeutic effect on DSS-induced colitis in mice.

## 2 Materials and methods

### 2.1 Materials

Mesalazine (MSZ) and CAP (Mw 2534.12) were purchased from Sigma-Aldrich (St. Louis, MO, United States). Mouse interleukin-6 (IL-6) and IL-1β ELISA Kits were purchased from mlbio (Shanghai, China).

### 2.2 Synthesis of rough hollow silicon (RHS) nanoparticles

RHS nanoparticles (RHS-NPs) were synthesized by a surfactant-free one pot process according to method reported by Song et al. with some modifications (Song et al., 2017). First, 0.2 g of resorcinol and 0.28 mL of formaldehyde (37 wt%) were added to the solution consisting of 3.0 mL of ammonia aqueous solution (28 wt%), 10 mL of deionized water and 70 mL of ethanol and stirred for 6 h at room temperature. Immediately, after the addition of 0.4 g of resorcinol and 0.56 mL of formaldehyde (37 wt%), 0.6 mL of TEOS was dropped into the solution and stirred for 8 min. The mixture was stirred for an additional 2 h at room temperature, and then transferred to a Teflon-lined autoclave and subjected to a hydrothermal treatment at 150°C for 24 h. The resultant composites were gathered through centrifugation, ethanol washing, and drying at 50°C. Finally, RHS-NPs were obtained after calcination at 550°C for 5 h in air.

### 2.3 Loading of MSZ and coating of CAP

5.0 mL of MSZ was dissolved in 2.0 mL of water. 5.0 mg of RHS nanoparticles were added to the solution and the suspension was stirred at room temperature for 48 h. The MSZ molecules could be adsorbed in the hollow structure. The as-prepared MSZ-loaded RHS nanoparticles (MSZ@RHS) were collected by centrifugation. The amount of the adsorbed guests was determined from the difference between the initial amount of MSZ by measuring the UV absorbance from the supernatant liquid at 298 nm quantified from a standard curve. Finally, MSZ@RHS were incubated in CAP solution (2 mg/mL, pH 6) for 20 min and washed 3 times with 0.05% NaCl to obtain MSZ@PRHS.

### 2.4 Characterization of RHS and PRHS nanoparticles

The structural shape and size of RHS and PRHS were characterized by scanning electron microscopy (SEM, Hitachi S-4800, Japan) at 20 kV and transmission electron microscopy (TEM, JEOL JEM-2100F, Japan) at 200 kV. Nitrogen sorption isotherms were measured at 77 K with ASAP 2420 and Micromeritcs Tristar 3020 analyzer (United States). Before measurements, the samples were degassed in vacuum at 180°C for 12 h.

The Brunauer–Emmett–Teller (BET) method was utilized to calculate the specific surface areas (SBET) using adsorption data in a relative pressure range from 0.05 to 0.2. The pore size distribution was obtained by applying proper nonlocal density functional theory (NLDFT) methods from the adsorption branch of isotherms.

The particle size distributions of RHS and PRHS nanoparticles in both water, 10 mM PBS and cell medium were measured by dynamic light scattering (DLS) analysis using a Zetasizer Nano-ZS from Malvern Instrument. The particle stability was tested through DLS analysis of RHS and PRHS suspension after incubating for 1, 24 and 48 h. The zeta potential of the RHS and PRHS nanoparticles were measured using a Zetasizer Nano-ZS.

The distribution of silica nanoparticles was determined by measuring silicon content in different organs by inductively coupled plasma atomic emission spectrometry (ICP) using an IRIS Advantage Duo ER/S (Thermo Fisher Scientific).

Fourier transform infrared (FTIR) spectroscopy was used to characterize the MSZ@PRHS nanoparticles in the solid state. FT-IR spectra were recorded on a Nicolet 6700 FT-IR spectrometer (Thermo Fisher Scientific) with scanning scope ranged from 4000 to 400 cm^–1^. Potassium bromide palettes were used for all spectra.

### 2.5 MSZ drug release

To study the effect of pH on drug release rate, MSZ@RHS and MSZ@PRHS were studied using simulated intestinal fluid (SGF; 16.4 mL of dilute hydrochloric acid was added into 800 mL of water, and then 20 g of pepsin was added, shake well and constant volume to 1,000 mL), simulated colon fluid (SCF; mlbio, Shanghai, China) and simulated gastric fluid (SIF; 6.8 g of dihydrogen phosphate was added into 500 mL of water. The pH value was adjusted to 4.5 with 0.1 mol/L sodium hydroxide solution, 10 g of pancreatin was added and constant volume to 1,000 mL). Briefly, 22.5 mg of MSZ@RHS and MSZ@PRHS nanoparticles were suspended in 50 mL of SGF agitated (60 rpm) at 37°C for 2 h, 50 mL of SIF for 2–6 h, and 50 mL of SCF for 6–18 h. At predetermined time intervals, 500 uL of the sample was collected from the solution and centrifuged at 45,000 x g at 4°C for 30 min. Then, the drug content in the supernatant was analyzed by high-performance liquid chromatography (HPLC) and the pellet was redispersed in the release buffer solution with 500 μL of fresh medium to avoid the loss of nanoparticles. The HPLC system (Shimadzu, Tokyo, Japan) used was equipped with an autosampler processor, SPD-20A ultraviolet (UV) detector, and C18 column (5 μm, 250 mm × 4.6 mm). The mobile phase consisting of a mixture of methanol: H2O (containing 3.6% glacial acetic acid) (73:27, v/v) was pumped at a flow rate of 1 mL/min. The sample injection volume was set at 20 μL and the UV detector was set at 298 nm. The retention time was 7.5 min at 25°C.

### 2.6 Cytotoxicity assay

The cytotoxicity of RHS and PRHS nanoparticles were tested in human colonic epithelial cells NCM460 by CCK-8 assay. NCM460 cells were seeded in a 96-well cell culture plate with a density of 1 × 10^4^ cells per well. After culture for 24 h, cells culture medium was then replaced by fresh 1,640 medium containing 10% FBS, where varied dosage of RHS and PRHS nanoparticles were added to each well. After incubation at 37°C for 48 h, 10 μL of CCK-8 solution was added to each well. Plates were then incubated in the culture oven for 2 h. Then absorbance readings were measured at wavelength of 540 nm using a microplate reader and background absorbance of media was subtracted. All experiments were performed in triplicate for each group. The statistical data are shown as mean +SD.

### 2.7 DSS-induced mouse model of colitis and treatment

C57B/6 female mice (8 weeks, SPF) were purchased from Charles River (Beijing, China). 27 mice were administered with 3.0% (w/v) DSS solution (MP Biomedicals, California, United States), added in their drinking water for 5 days. The five controlled mice received clean water for 5 days. After induction of colitis, DSS water was replaced with normal tap water and drug treatment was started. The mice were randomly divided into five groups: 1) Control group; 2) DSS group; 3) MSZ group; 4) MSZ@RHS group; 5) MSZ@PRHS group and each group were administrated by intragastrical gavage with 7.4 mg/kg free MSZ, MSZ@RHS or MSZ@PRHS once a day for 7 days, respectively. DSS group mice received saline. All mice were anaesthetized and fur was removed from the intestinal region. Mice were monitored daily for body weight, food intake, water intake, rectal bleeding, survival and stool consistency. For the histological scoring, colons were embedded in OCT compound. Specimens were sectioned into 5 μm slices and stained using the Hematoxylin and Eosin (H&E) method. All animal procedures were approved by the Institutional Animal Care and Use Committee in accordance with the First Affiliated Hospital of Shandong First Medical University and Shandong Provincial Qianfoshan Hospital.

### 2.8 Macroscopic grading of colitis

The macroscopic assessment of colitis was performed based on disease activity index (DAI), colon length and spleen weight of mice as previously reported (He et al., 2016). Clinical scores were evaluated by an independent investigator who was blinded to the experiment.

### 2.9 Bio-distribution of RHS and PRHS nanoparticles in mice gastrointestinal tract (GIT)

The bio-distribution of RHS and PRHS nanoparticles in the different segments of the GIT in colitis-induced mice was studied. Briefly, mice were randomly divided into the following two groups (*n* = 3 mice per group): RHS and PRHS group. Nanoparticle suspensions (ICG dye was used as fluorescent probe) were prepared and administered by intragastrical gavage to mice (7.4 mg/kg of body weight). The bio-distribution of RHS and PRHS nanoparticles in mice GIT was also studied using an *in vivo* imaging system (IVIS) (FOBS; Nanoscience, South Korea). After 24 h of administration, the whole GIT of mice was stripped for *ex vivo* fluorescence imaging.

### 2.10 Measurement of MPO activity and pro-inflammatory cytokine levels

MPO activity in the colon tissues was measured by following a previously described method. Briefly, 50 mg of the tissue sample was homogenized in 2 mL of ice-cold 50 mM phosphate buffer (pH 6.0) containing 0.5% hexa-decyltrimethyl ammonium bromide at 30 Hz and 4°C for 4 min. The homogenate was then centrifuged at 13,400 xg for 6 min at 4°C. The supematant was then collected and added to 0.0167% o-dianisidine hydrochloride (o-dianisidine) solution and 0.0005% hydrogen peroxide (H_2_O_2_), and the change in absorbance was measured at 450 nm at intervals of 30 s. The o-dianisidine solution was prepared by dissolving 16.7 mg of o-dianisidine in 90 mL of distilled water and 10 mL of 50 mM phosphate buffer. MPO activity was calculated as a unit of MPO per mg of tissue. One unit of MPO was defined as the amount needed to degrade 1 μmol H_2_O_2_ per min.

The concentration of interleukin-6 (IL-6) and IL-1 1beta (IL-1β) were quantified in the colon samples according to manufacturer’s description (mlbio, Shanghai, China). Briefly, 50 mg of the colon tissues were homogenized in 1 mL of protease inhibitor cocktail and lysis buffer solution for 5 min. The homogenate was centrifuged at 3300 *g* for 5 min at 4°C and the supernatant was used to quantify IL-6 and IL-1β by sandwich-type enzyme-linked immunosorbent assay.

### 2.11 Biological safety testing

For liver toxicity, alanine aminotransferase (AST) and aspartate aminotransferase (ALT) activities were determined as described previously (Bonfanti et al., 2020). Similarly, for kidney toxicity studies of blank nanoparticles RHS and PRHS, blood urea nitrogen (BUN) and creatinine were also estimated.

### 2.12 Statistical analysis

Results were expressed as mean ± standard deviation. All data were analyzed using one way analysis of variance followed by Tukey’s test. Values of *p* < 0.05 were considered as significant. All the statistical analyses were performed using graph pad prism five software (Graph Pad Software, Inc., San Diego, CA, United States).

## 3 Results and discussions

### 3.1 Preparation and characterization of the RHS and PRHS nanoparticles

**FIGURE 1:**
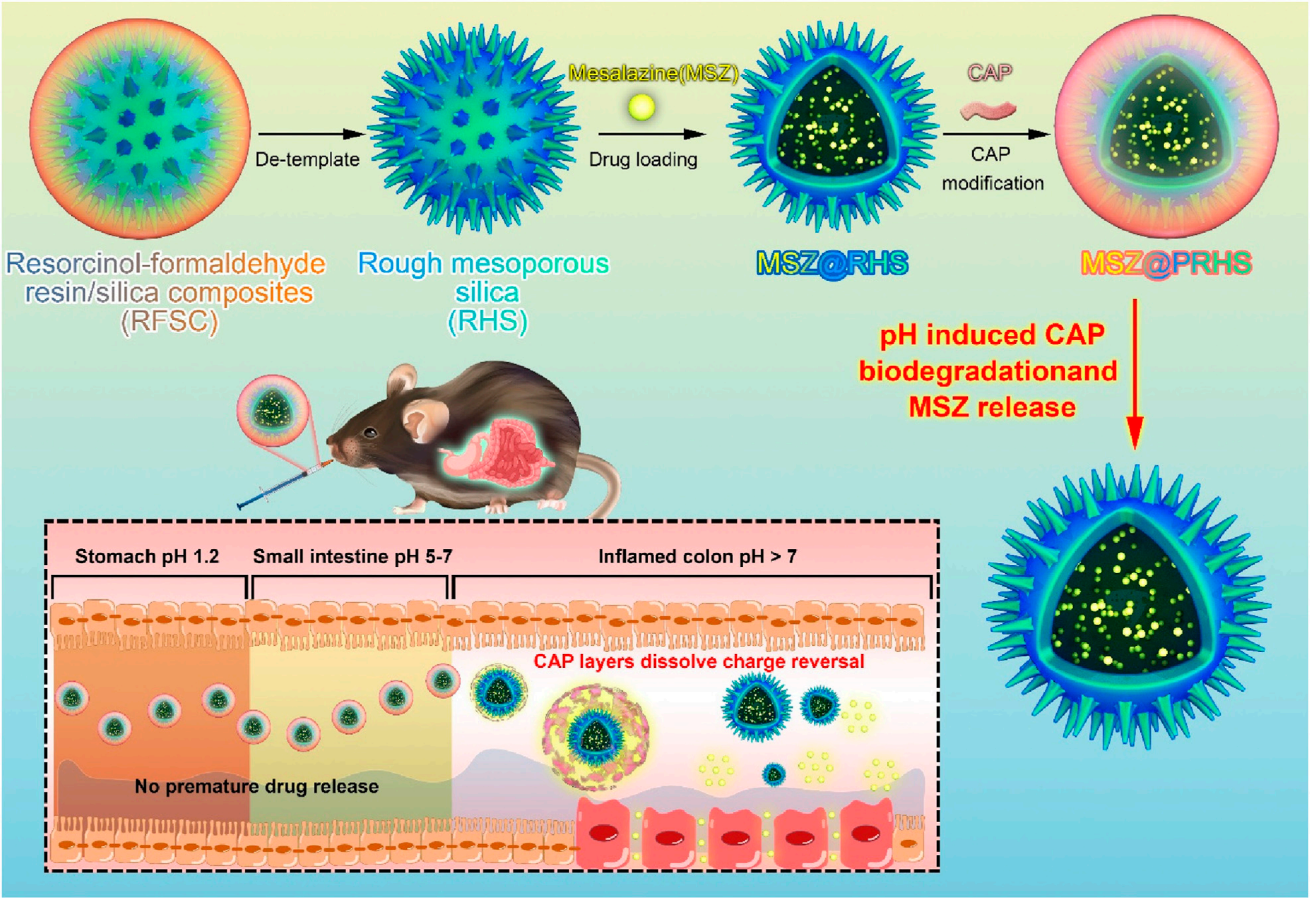
Schematic design of MSZ@PRHS composite nanoparticles targeting the colon.

**FIGURE 2:**
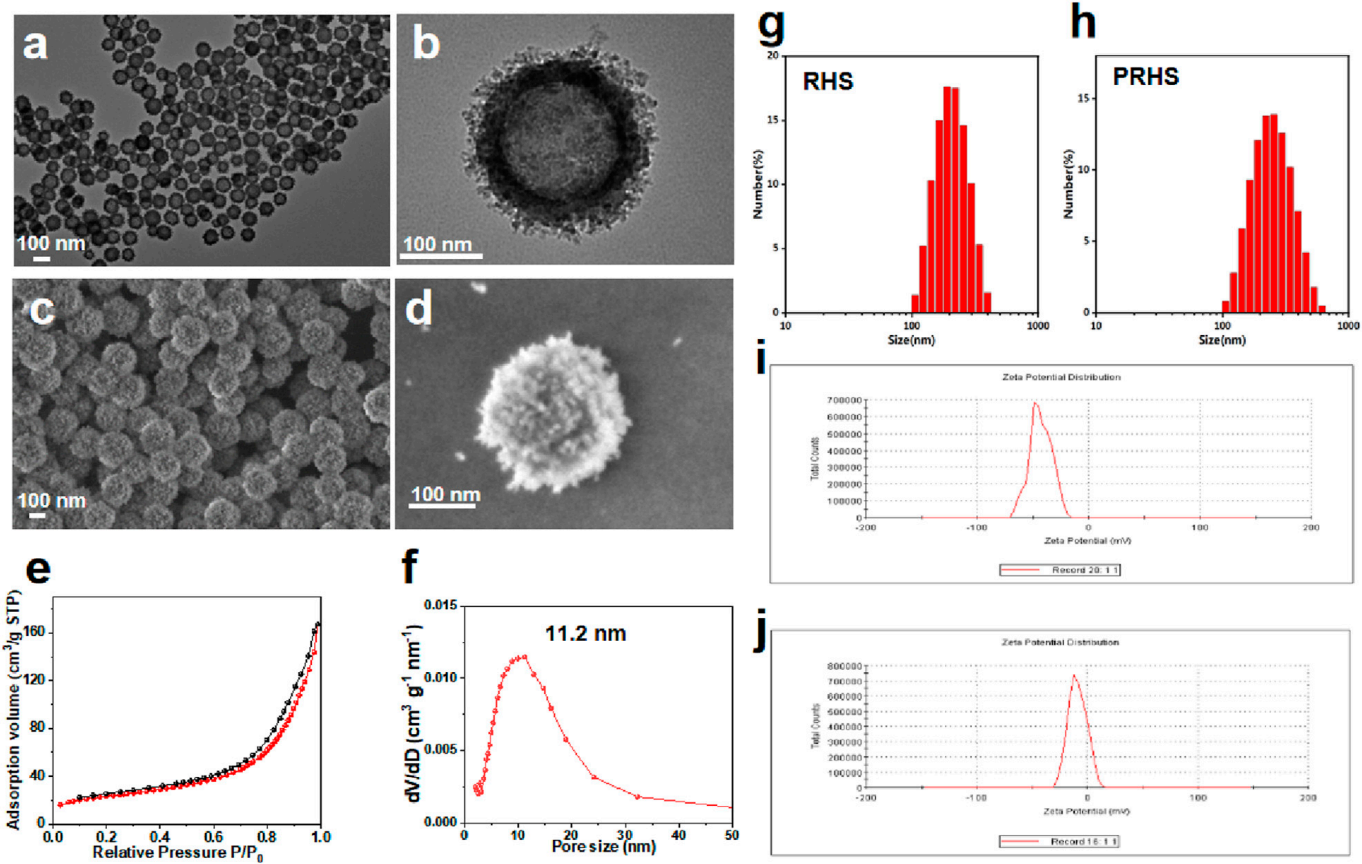
Characterization of RHS nanoparticles. **(A, B)** Structural characterization of RHS by TEM; **(C, D)** Structural characterization of RHS by SEM; **(E, F)** Nitrogen adsorption and desorption isotherms **(E)** and BJH pore size distribution curves derived from adsorption branches **(F)** of RHS nanoparticles; Size distribution of **(G)** RHS and **(H)** PRHS nanoparticles by DLS analysis; Zeta analysis of **(I)** RHS and **(J)** PRHS nanoparticles.

The synthesis of RHS nanoparticles was accomplished using a surfactant-free onepot process (Song et al., 2016; Song et al., 2017), that is, by assembling phlorophenol-formaldehyde (RF) resin and silica primary particles under Stöber synthesis conditions (Figure 1). Transmission electron microscopy (TEM) images of the obtained RHS nanoparticles clearly show an inner hollow structure and the obvious rough surface morphology of the outer surface with uniform particle size of ∼225 nm (Figure 2A). At higher magnification (Figure 2B), it is clear that each RHS nanoparticle has an internal hollow cavity (145 nm in diameter) and a shell (30 nm in thickness) covered with nano-silica spikes. The DLS result further confirm the nanospheres are monodispersed with a polydispersity index (PDI) of 0.245, and the hydrodynamic particle size is measured to be 224 nm, which is similar to the particle size obtained from TEM images (Figure 2G). The particle sizes of silica nanoparticles measured from DLS are relatively larger than those determined by TEM analysis due to the hydration of the silica surface by surrounding water molecules (Keasberry et al., 2017). Scanning electron microscopy (SEM) images further revealed nanoscale surface roughness with densely distributed silica spikes on the surface (Figures 2C, D). To characterize the porosity of the RHS surface, nitrogen sorption analysis was performed. The nitrogen adsorption and desorption isotherm of RHS shows a typical type IV isotherm (Figure 2E). The corresponding Barrett–Joyner–Halenda (BJH) pore size distribution curve (Figure 2F) derived from adsorption branch exhibits a relatively broad peak centered at 11.2 nm. The Brunauer–Emmett–Teller (BET) surface area and the total pore volume of RHS are 83 mg^2^/g and 0.01148 cm^3^/g/nm, respectively. RHS nanoparticles have a large specific surface area and abundant mesopores, which can be used for loading therapeutic drugs.

**FIGURE 3:**
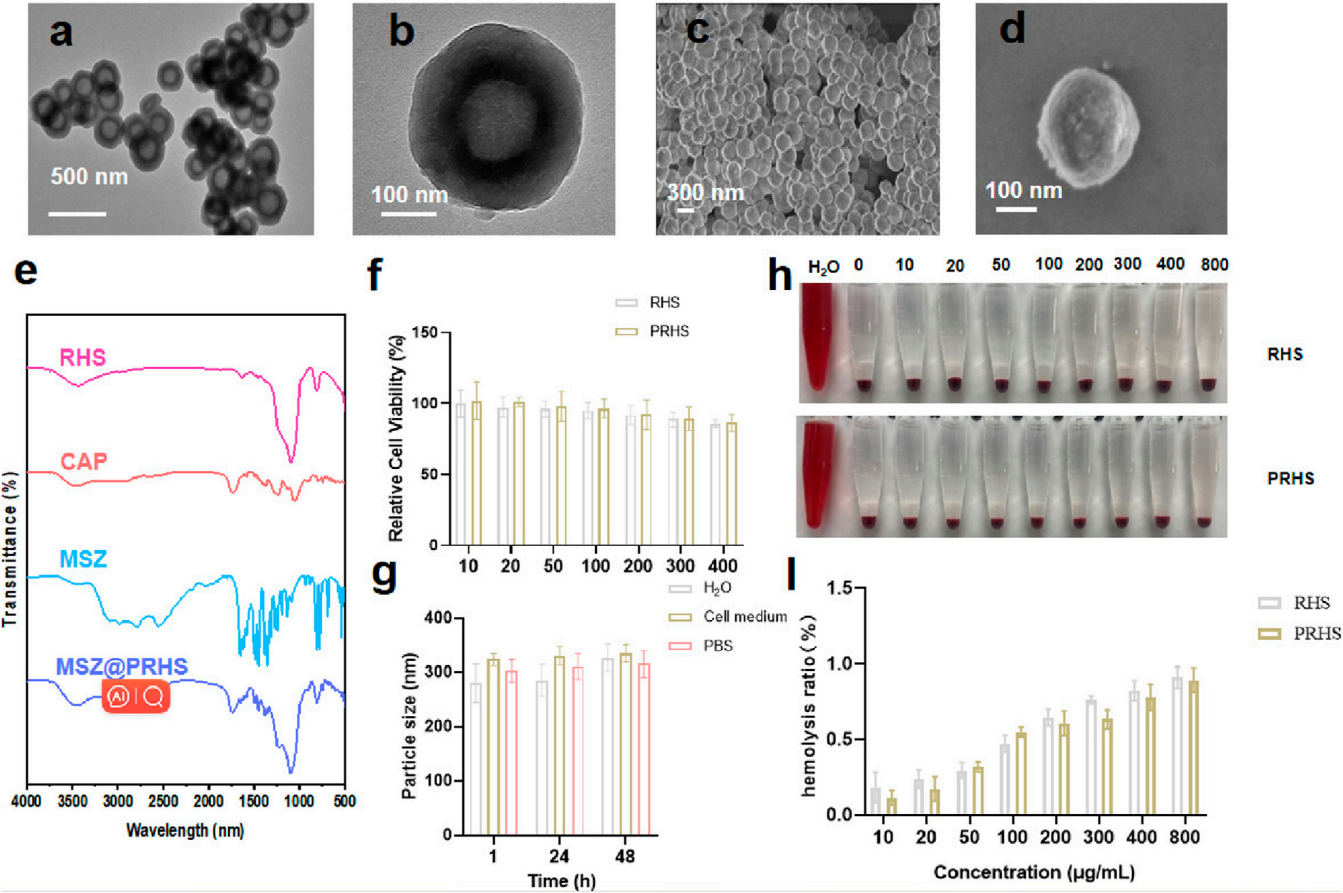
Characterization of PRHS nanoparticles. **(A, B)** Structural characterization of PRHS by TEM; **(C, D)** Structural characterization of PRHS by SEM; FTIR spectrum of RHS, CAP polymer, free MSZ, and MSZ@PRHS nanoparticles **(E)**; **(F)** Viability of NCM460 cells treated with RHS or PRHS nanoparticles at concentrations ranging from 0 to 400 μg/mL for 24 h; **(G)** Stability of RHS or PRHS nanoparticles in different media detected by DLS; **(H, I)** Hemolysis images and rate of RHS or PRHS nanoparticles.

Studies have shown that cellulose acetate phthalate (CAP) can protect encapsulation material from gastric acid in the stomach and release encapsulation material under slightly neutral to alkaline conditions in the gut (Huang et al., 2012; Hanafi et al., 2013). We coated the CAP uniformly on the surface of the RHS by a coating technique according to previous reports (Levine et al., 1987; Oshi et al., 2020). To verify that CAP was successfully coated on the surface of RHS, Zeta potential was detected before and after coating, as shown in Figures 2I, J (from −21.5 mV to −8.5 mV). Further structural observations of PRHS nanoparticles were performed using TEM analysis (Figures 3A, B). The dark gray region with high contrast indicates the RHS core, while the shallower and deeper parts surrounding the core confirm the presence of the CAP layer shell. The diameter of the RHS core is about 115–165 nm, while the thickness of the CAP layer shell is about 60–80 nm. In general, it has been reported that the core-shell nanoparticles with thick shell have the advantage of achieving a sustained drug release from their inner cores (Jahns et al., 2020). The DLS result further confirm the nanospheres are monodispersed with a polydispersity index (PDI) of 0.257, and the hydrodynamic particle size is measured to be 255 nm, which is similar to the particle size obtained from TEM images (Figure 2H). SEM result showed that the PRHS nanoparticles were spherical, uniform in size and the CAP shell was completely wrapped (Figures 3C, D), which was consistent with the above results. The FTIR results were shown in Figure 3E, indicating that RHS indicated successful encapsulation and loading of CAP polymer with MSZ.

### 3.2 pH-dependent drug release

**FIGURE 4:**
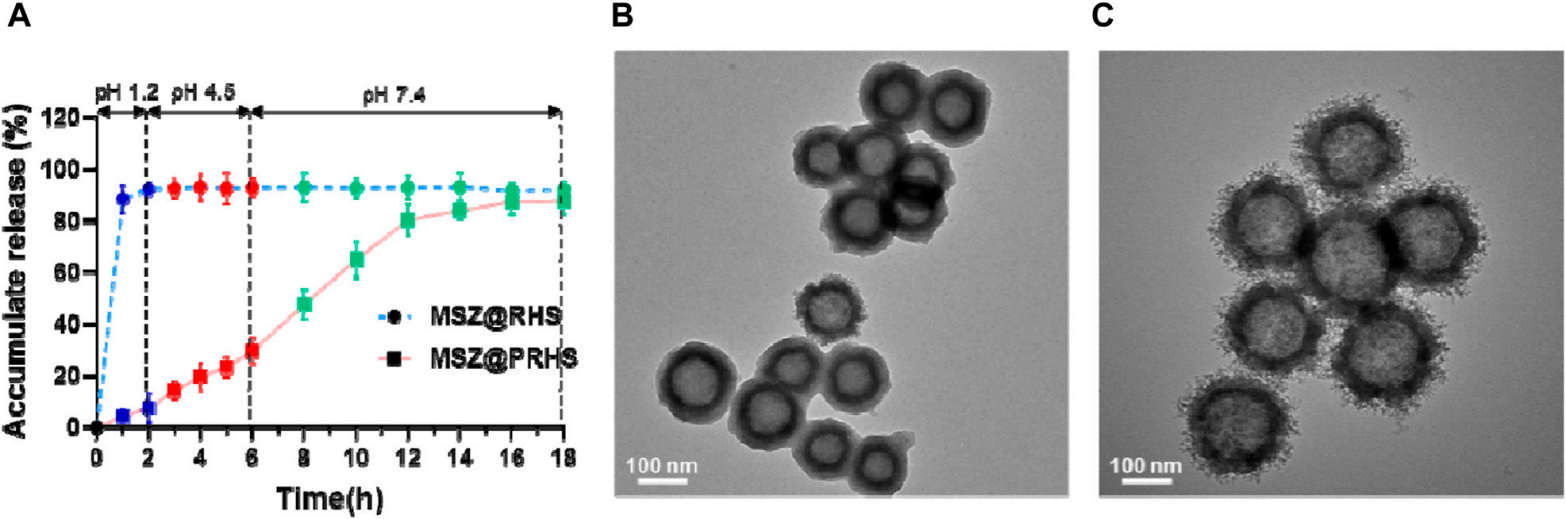
Drug release of the nanoparticles based on pH variation. **(A)** pH-dependent drug release from MSZ@RHS and MSZ@PRHS nanoparticles; **(B)** Structural characterization of PRHS by TEM with SGF solution; **(C)** Structural characterization of PRHS by TEM with SCF solution.

After oral administration, anti-inflammatory drugs first pass through the stomach (pH: 1.2, 1–2 h), then through the small intestine (pH: 4.5, 3–6 h), and finally to the large intestine (pH: 7.2, 12–24 h). Therefore, we investigated the sustained release properties of MSZ@PRHS in dissolution medium mimicking gastric fluid (SGF, pH 1.2), intestinal fluid (SIF, pH 4.5) and colon fluid (SCF, pH 7.2). MSZ@RHS had a encapsulation efficiency of 93.5% and a drug loading capacity of 35.6%, while MSZ@PRHS had a encapsulation efficiency of 91.6% and a drug loading capacity of 32.4%. As shown in Figure 4A, no bursting MSZ drug release occurred in either SIF or SGF solutions, because CAP dissolution occurred only when pH was greater than 6. After incubation with SGF for 2 h, MSZ@PRHS released only 7.68% of MSZ, and 4-h incubation in simulated intestinal fluid at pH 6.8 resulted in a 29.1% release. On the other hand, after incubation in SCF, 92.9% of loaded MSZ was released from NPs within 8 h. The results showed that MSZ@PRHS nanoparticles can release MSZ in response to the colonic environment consistent with previous reports (Oshi et al., 2020). Then, TEM images and particle size distributions of MSZ@PRHS in different simulation solutions were tested (Figures 4B, C). In SGF solutions, almost all the CAP shell of this nanoparticle is intact, whereas after incubation in SCF for 2h the material exhibits a rough surface structure of RHS, which is due to the dissolution of CAP. These results indicate that MSZ@PRHS nanoparticles are promising delivery vectors for MSZ in the colon due to their unique properties.

### 3.3 *In Vitro* biological properties of RHS and PRHS nanoparticles

The RHS and PRHS nanoparticles were incubated with human normal intestinal epithelial cell NCM-460 at different concentrations (10, 20, 50, 100, 200, 300 and 400 μg/mL) for 24 h. Then, the cell counting kit-8 (CCK-8) assays were carried out to detect the relative cytotoxicity, as shown in Figure 3F, no significant decrease in cellular viability was observed, indicating the ignorable cytotoxicity of RHS and PRHS nanoparticles. To verify the stability of PRHS nanoparticles under physiological conditions, the nanoparticles were placed in water (H_2_O), phosphate buffer (PBS) and culture medium for 1, 24, and 48 h, and then the size of nanoparticles was determined via DLS. The results suggested that the average size of PRHS nanoparticles retained about 300 nm, indicated the strong stability of the PRHS nanoparticles (Figure 3G). Additionally, the hemocompatibility of RHS and PRHS nanoparticles were preliminarily assessed by the hemolytic assay. Red blood cells were exposed to RHS and PRHS nanoparticles and the absorbance at 540 nm of the collected supernatants was measured to determine the hemolytic percentage. As presented in Figure 3H, the supernatants of all samples were completely transparent, which corroborated that the structural integrity of red blood cells maintained well regardless of the concentration of RHS and PRHS nanoparticles. Moreover, the quantitative result of hemolytic percentage showed that no hemolytic effects occurred even at a high concentration of 800 μg/mL (lower than 5%) (Figure 3I).

Therefore, these results exhibited the excellent physiological stability and biocompatibility of RHS and PRHS nanoparticles and were capable of medical treatment application.

### 3.4 *In Vivo* biological distribution of RHS and PRHS nanoparticles

**FIGURE 5:**
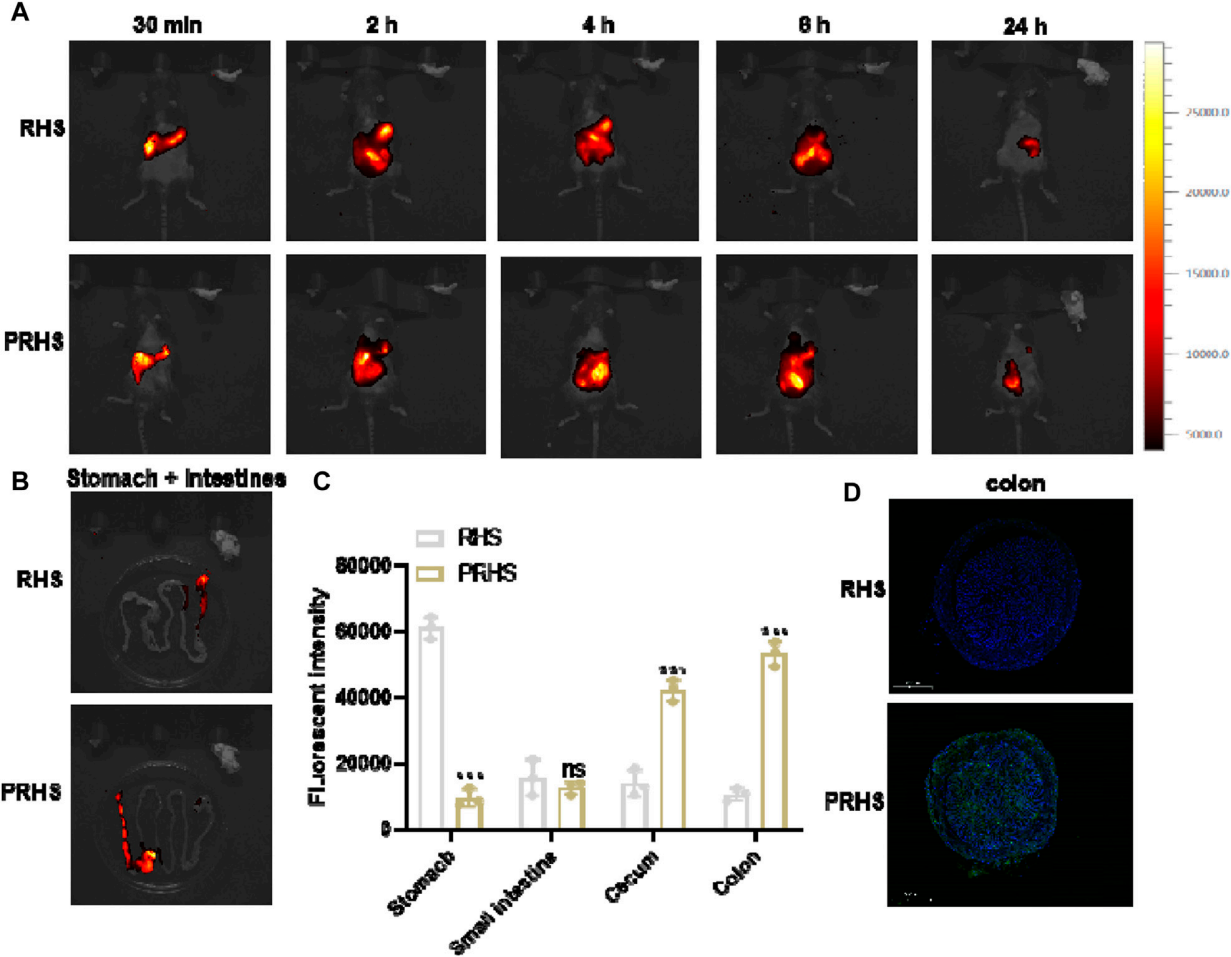
*In vivo* biological distribution of RHS and PRHS nanoparticles. **(A)** *In vivo* imaging system measurement of mice after oral administration with RHS-ICG or PRHS-ICG (100 mg/kg) for different time points. **(B)** Fluorescence images of stomachs and intestines were recorded at 24 h post gavage; **(C)** Quantification of the fluorescence intensities of the stomach, small intestine, cecum, and colon by *in vivo* imaging system; **(D)** Fluorescence intensity in the colon of mice 24h after oral administration of RHS-FITC or PRHS-FITC (100 mg/kg). Results are expressed as the mean value ±SD of data obtained from separate experiments. ***p < 0.001, ns indicates no statistical difference, compared with RHS group.

We next explored the *in vivo* fate of RHS and PRHS after oral administration, respectively, by tracing and visualizing ICG signals in C57 mice, recorded using an *in vivo* imaging system. Compared with the RHS group, the PRHS group produced a stronger and longer red fluorescence distribution, especially in the abdomen of the mice, which lasted for more than 24 h (Figure 5A). The stronger fluorescence of PRHS in live mice and in the intestine may be due to increased intestinal absorption of CAP and enhanced retention of the drug contained. After 24 h, the stomach and intestinal organs were taken for further imaging and quantification. RHS A clear fluorescence signal was observed in the stomach of mice and a slight fluorescence signal was observed in the small intestinal segment, but not in the colonic segment. PRHS Weak fluorescence signals were detected in the stomach, small intestine, and caecum (non-colon), whereas there was a distinct fluorescence signal in the colonic segment (Figures 5B–D). These results provide evidence that RHS is solubilized in the stomach and small intestine before reaching the colon, in contrast to PRHS, which is able to reach the colon for efficient distribution. This is due to the free form of carboxyl group in the CAP polymer, which can be salted in alkaline environment (pH > 6), and the rough surface structure of RHS can reside in the colon for a longer time when it is exposed to the colon (Jagdale and Chandekar, 2017; Singh et al., 2022).

### 3.5 Treatment efficacy of MSZ@PRHS in IBD mice

**FIGURE 6:**
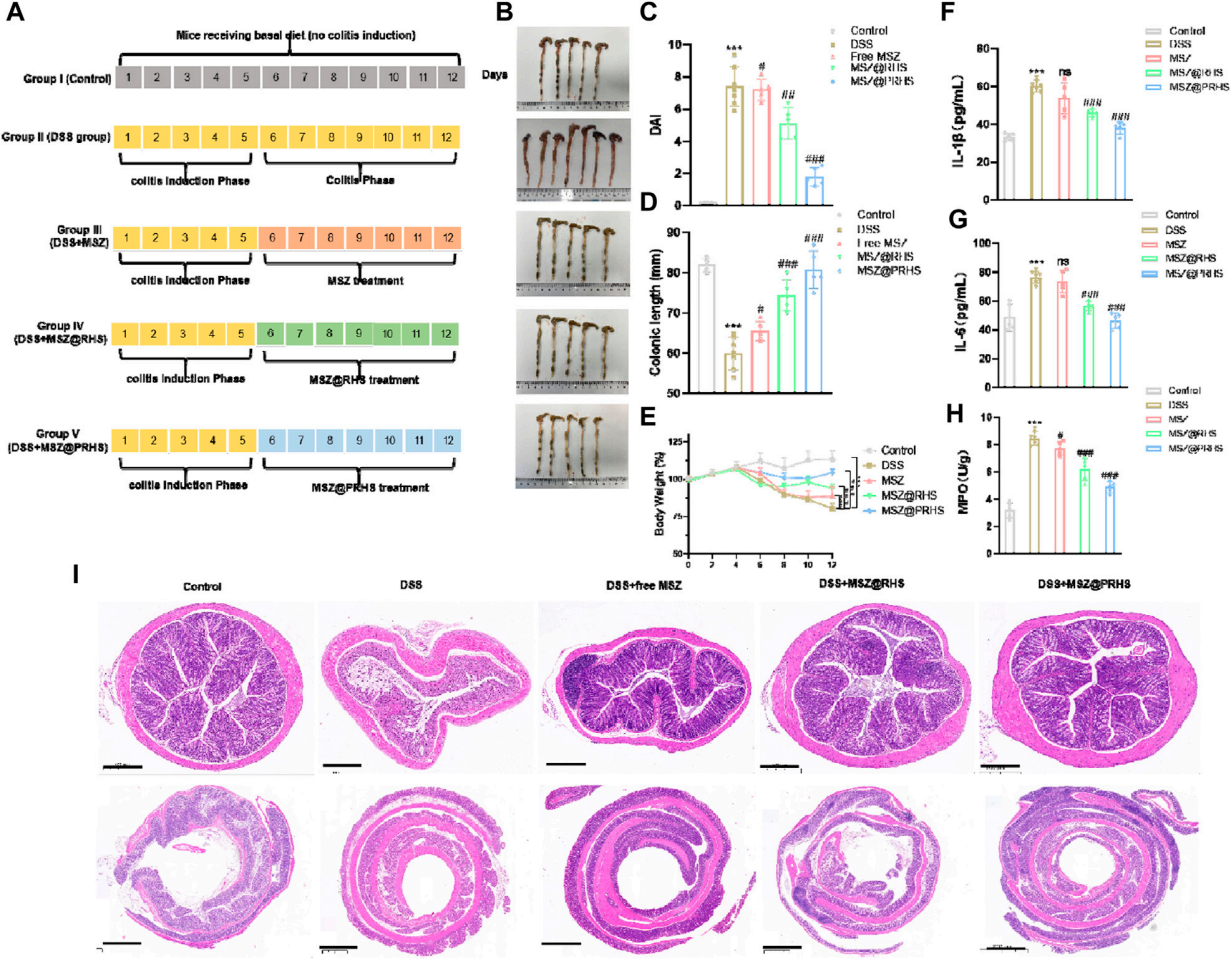
Treatment efficacy of MSZ@PRHS in IBD Mice. **(A)** Experimental design for the treatment of a murine model of colitis induced by DSS; **(B)** Photographs of colons after treatment; **(C)** Disease activity index (DAI); **(D)** colon length; **(E)** Bodyweights of mice at the indicated time points; **(F, G)** Concentrations of **(F)** IL-1β and **(G)** IL-6 in serum collected from the treated mice. **(H)** Expression levels of MPO in colons after treatment. Treatment Efficacy of MSZ@PRHS in IBD Mice; **(I)** MSZ@PRHS eliminated inflammation and improved the morphology of the colon. Results are expressed as the mean value ±SD of data obtained from separate experiments. ***p < 0.001, compared with Control group; ns indicates no statistical difference, #*p* < 0.05, ##*p* < 0.01 and ###*p* < 0.001, compared with DSS group.

After clarifying the targeting effect of MSZ@PRHS on the colon, we further examined the therapeutic effect of MSZ@PRHS on DSS-induced colitis in mice. The characteristics of DSS-induced colitis in mice are similar to those in humans, including weight loss, bloody diarrhea, ulcer formation, and epithelial cell loss (Li et al., 2022)。DSS-induced colitis mouse model was established by feeding with 3% (wt%) DSS instead of water for five consecutive days, and then DSS was replaced by free MSZ (7.4 mg/kg), MSZ@RHS and MSZ@PRHS (equal amount of MSZ). Macroscopic changes in DAI, colon length and body weight were measured after 7 days of treatment. The specific modeling and drug administration diagram was shown in Figure 6A. DAI was used to indicate the severity of colitis, and the results were shown in Figure 6C. Compared with the control group, the DAI values of DSS-induced colitis mice were significantly increased, indicating high severity of colitis. Both the free MSZ and MSZ@RHS groups showed slight decrease in DAI, while the MSZ@PRHS group showed significantly lower DAI, indicating lower severity of colitis. In addition, we euthanized all mice at the end of treatment and measured the length of the mouse colon to assess the severity of colitis (Figures 6B, D). The colon lengths of the MSZ@PRHS treated mice were similar to that of the control mice (∼82 mm), which approximately ∼80.8 mm. The colon lengths of free MSZ (∼65.6 mm) and MSZ@RHS (∼70 mm) treated mice were significantly shortened, similar to that in the colitis group (∼55 mm), indicating severe colitis. We also found that only MSZ@PRHS treatment was effective in restoring body weight in DSS mice (Figure 6E). In addition, among all treated mice, mice given MSZ@PRHS presented the lowest concentrations of IL-1β and IL-6, indicating the ability to attenuate the inflammatory response in DSS mice (Figures 6F, G).

Myeloperoxidase (MPO) is an abundant neutrophil granule that plays an important role in killing bacteria and promoting inflammatory tissue damage by producing the strong oxidant hypochlorous acid (Aratani, 2018). Therefore, it is considered to be an important marker for detecting the severity of mucosal neutrophilic infiltration and colitis. Hence, MPO levels were measured activity in colon samples of all mice groups. MPO levels were also significantly reduced in the colon of MSZ@PRHS treated mice compared with all treated mice (Figure 6H). The colon section of healthy mice had intact mucosa and no signs of mucosal inflammation, i.e., intact epithelium, no edema, no infiltration of inflammatory cells, neutrophils, and macrophages. In contrast, the colitis of mice with DSS induced colitis exhibited severe mucosal inflammatory histology, namely, epithelial damage, edema, and infiltration of the mucosa by neutrophils and macrophages. In addition, only a slight improvement in the histological features of colitis was observed in mice treated with free MSZ and MSZ@RHS. As expected, severe histological features of colitis improved significantly after treatment with MSZ@RHS, with reformation of mucosal epithelial cells, absence of edema, and lower rates of infiltration of neutrophils and macrophages into the mucosa (Figure 6I). Although the use of either free MSZ or MSZ@RHS produced beneficial effects, the use of MSZ@PRHS eliminated inflammation and improved the morphology of the colon.

### 3.6 *In vivo* biosafety evaluation of RHS and PRHS nanoparticles

In addition, the potential *in vivo* toxicity of the RHS and PRHS nanoparticles was investigated in terms of blood chemistry and histological evaluation. The dispersion of the RHS and PRHS nanoparticles were injected into healthy C57 mice at dose of 20 mg/kg by gavage. Then, the blood of the experimental mice was collected from the eyeballs after 1 month injection. The samples were sent for serum biochemistry examination. As shown in Supplementary Figures S1A–C, the serum parameters of the RHS and PRHS nanoparticles treated mice exhibited no abnormal values as compared with the control group, involving the liver function indexes of alanine aminotransferase (ALT) and aspartate aminotransferase (AST), the kidney function markers of blood urea nitrogen (BUN) and creatinine (CREA). Moreover, the major organs (heart, liver, spleen, lung, and kidney) of the treated mice were excreted, sliced, and stained with hematoxylin and eosin (H&E) for histological evaluation after 2 weeks of intravenous injection for the RHS and PRHS nanoparticles. The results also indicated that no perceptible tissue damage or inflammatory lesion was observed in all tissue samples, which is similar with the control group (Supplementary Figure SD). Thus, the above results demonstrated that the developed the RHS and PRHS nanoparticles have negligible toxicity *in vivo*, permitting its safe applications for therapy.

## 4 Conclusion

In this study, we developed colon-targeted composite nanoparticles based on rough mesoporous silica (RHS) and pH-responsive outer membrane of CAP for specific drug delivery to the colon. *In vitro* release study showed that the release of MSZ@PRHS was pH-dependent. Biodistribution studies showed that PRHS had a high colonic distribution and long-term residence in the colon of mice. MSZ@PRHS showed a stronger therapeutic effect on DSS-induced colitis in mice. In conclusion, the drug delivery system developed in this study has great potential for application in colon-targeted colitis therapy. In addition, we will further investigate the influence of MSZ@PRHS on intestinal flora in the following studies.

## Data Availability

The raw data supporting the conclusion of this article will be made available by the authors, without undue reservation.
