# Supplementary material for: pH-responsive interface conversion efficient oral drug delivery platform for alleviating inflammatory bowel disease
Source: Front Chem. 2024 Mar 12;12:1365880. doi: 10.3389/fchem.2024.1365880 (PMC10963395; doi:10.3389/fchem.2024.1365880)
Supplement: Supplementary file 1 [file DataSheet1.docx]

pH-responsive interface conversion efficient oral drug delivery platform for alleviating inflammatory bowel disease

# Supplementary Figures


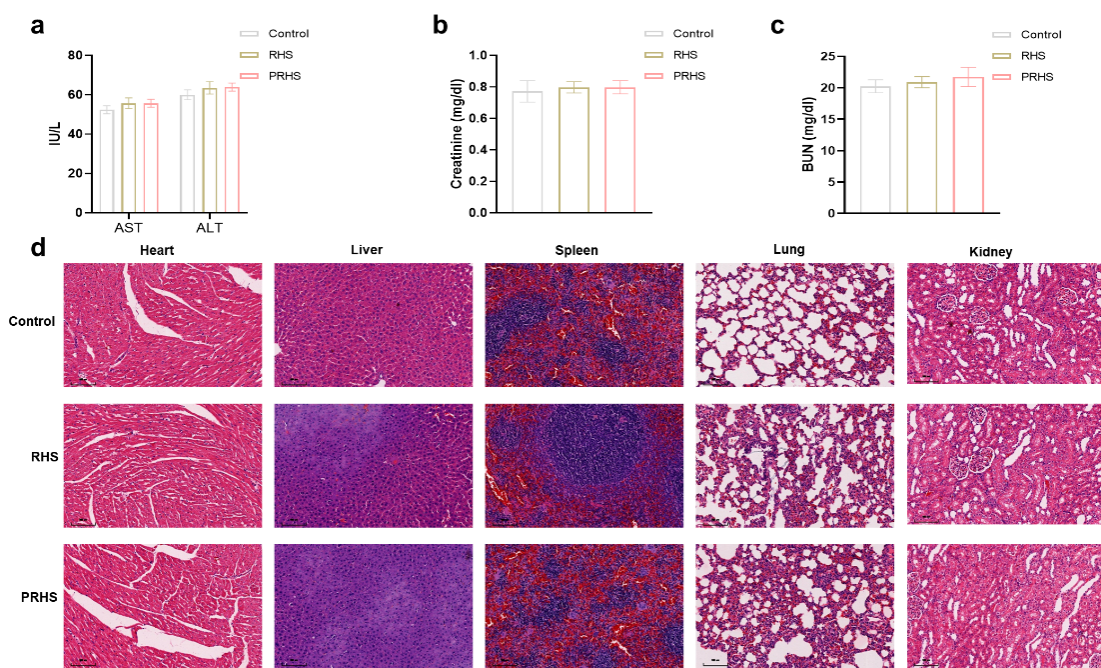
**Figure S1.** Biosafety of RHS and PRHS nanoparticles in vivo. Detection of serum (a) ALT and AST, (b) CREA and (c) BUN levels after the acute toxicity test. n=3 mice per group. (d) Histopathological examination of vital organ tissues after oral of RHS and PRHS nanoparticles once daily for one month by H&E staining. Scale bars, 200 μm. n = 3 mice per group.
